# The Healthy Brain 9 (HB9): A New Instrument to Characterize Subjective Cognitive Decline

**DOI:** 10.1002/alz70857_099870

**Published:** 2025-12-24

**Authors:** James E Galvin, Katherine C Almonte, Andrea D Buehler, Yolene M Caicedo, Conor B Galvin, Willman B Jimenez, Mahesh S Joshi, Nicole Mendez, Mary Lou E Riccio, Marcia I Walker, Michael J Kleiman

**Affiliations:** ^1^ University of Miami Miller School of Medicine, Boca Raton, FL, USA; ^2^ University of Miami Miller School of Medicine, Miami, FL, USA

## Abstract

**Background:**

Subjective cognitive decline (SCD) affects 10% of older adults ≥45y with a differential expression across differential ethnoracial groups: 9.3% of non‐Hispanic Whites, 10.1% of African Americans, and 11.4% of Hispanics. SCD may be a risk factor for future mild cognitive impairment (MCI) and dementia. We developed the Healthy Brain 9 (HB9) as a self‐report assessment of cognitive functioning and whether self‐recognized changes interfere with daily functioning in a diverse community‐based longitudinal cohort.

**Method:**

Consecutive participants (*n* = 357) enrolled in the Healthy Brain Initiative and underwent comprehensive baseline evaluations modelled on the UDS v3.0. The HB9 was validated against a comprehensive assessment of clinical, cognitive, functional and behavioral measures, structural MRI, and blood‐based biomarkers.

**Result:**

Participants had a mean age of 68.6 ± 9.8y, 69.9% were female, 62.3% had 16 or less years of education, and 40.3% were from ethnoracial minorities. The sample included 42.3% normal cognition, 24.9% SCD and 32.8% MCI. The HB9 showed small‐to‐moderate correlations with most clinical measures and moderate‐to‐large correlations with the AD8 and QDRS‐patient version, two measures of subjective cognitive functioning. The HB9 showed small‐to‐moderate correlations with neuropsychological tests suggesting that the HB9 was not measuring objective cognitive performance but rather the participants’ perception of their performance compared to how they thought they would have done in the past. The HB9 showed a weak correlation with NFL (*r* = 0.156, *p* = 0.008). The HB9 was weakly correlated with smaller hippocampal volumes and higher cortical atrophy scores. The HB9 worked equally well across different ethnoracial, sociodemographic, and diagnostic groups with similar performance in neurodegenerative vs non‐neurodegenerative MCI etiologies. Lower HB9 scores were associated with greater resilience, better physical performance, and less physical frailty. Higher HB9 scores were associated with more comorbid medical conditions, more mood symptoms, higher perceived stress, and poorer physical functionality. A cut‐off score of 3.5 had best discrimination of SCD (AUC 0.845; 95%CI: 0.800‐0.890).

**Conclusion:**

While not a definitive diagnosis, use of the HB9 as an assessment of SCD may help identify individuals in the early stages of a neurodegenerative disease who could benefit from further monitoring, potential interventions, and enrollment into clinical trials.

